# Sex Differentially Alters Secretion of Brain Extracellular Vesicles During Aging: A Potential Mechanism for Maintaining Brain Homeostasis

**DOI:** 10.1007/s11064-022-03701-1

**Published:** 2022-07-29

**Authors:** Yohan Kim, Rocío Pérez-González, Chelsea Miller, Michelle Kurz, Pasquale D’Acunzo, Chris N. Goulbourne, Efrat Levy

**Affiliations:** 1 Center for Dementia Research, Nathan S. Kline Institute, Orangeburg, NY 10962, USA; 2 Department of Psychiatry, New York University Grossman School of Medicine, New York, NY 10016, USA; 3 Alicante Institute for Health and Biomedical Research (ISABIAL) and Neuroscience Institute UMH-CSIC, 03550 Alicante, Spain; 4 Department of Biochemistry & Molecular Pharmacology, New York University Grossman School of Medicine, New York, NY 10016, USA; 5 Neuroscience Institute, New York University Grossman School of Medicine, New York, NY 10016, USA

**Keywords:** Extracellular vesicles, Microvesicles, Exosomes, Mitovesicles, Aging, Sex, Brain

## Abstract

Extracellular vesicles (EVs) in the brain play a role in neuronal homeostasis by removing intracellular material and regulating cell-to-cell communication. Given that sex and aging differentially modulate brain networks, we investigated sex-dependent differences in EV levels and content in the brain during aging. EVs were isolated from the brains of 3, 6, 12, 18, and 24 month-old female and male C57BL/6 J mice, and the levels of different EV species determined. While the number of plasma membrane-derived microvesicles and a subset of late endosomes-derived exosomes increased with age in the brain of female mice, no significant changes were seen in males. Mitochondria-derived mitovesicles in the brain increased during aging in both sexes, a change that may reflect aging-dependent alterations in mitochondrial function. These findings reveal enhanced turnover during aging in female brains, suggesting a mechanism for advantageous successful female brain aging and sex-depending different susceptibility to age-related neurodegenerative diseases.

## Introduction

Extracellular vesicles (EVs) are nanoscale phospholipid bilayer membrane-bound vesicles containing lipids, proteins, and RNAs that are released by cells into tissue extracellular space, biological fluids, and conditioned culture media [1]. We and others have found that brain EVs, vesicles derived from discrete intracellular compartments and secreted into the extracellular space, can be highly informative of cellular processes and disease states. Studies have shown that distinct subpopulations of EVs have unique molecular compositions [2, 3]. Microvesicles, derived from the plasma membrane and having a diameter of 100–1000 nm, show characteristics of the membrane and cytoplasm of the cell of origin and changes due to physiological and pathological conditions. Exosomes are generated as intraluminal vesicles (ILVs) through the invagination of the membrane of late-endosomes/multivesicular bodies (MVBs) in the endosomal-lysosomal pathway, having a diameter of 50—150 nm. They are informative of the health of the endosomal-lysosomal pathway and changes that occur in multiple disorders, including spinocerebellar ataxia [4] and aging-dependent neurodegenerative diseases, such as Down syndrome (DS), Alzheimer’s disease (AD) and Parkinson disease (PD) [5]. For example, the interrelationship between endosomal pathology and the level of release and content of exosomes was demonstrated in DS [6–8] and the brain of carriers of the apolipoprotein E4 allele, the major risk factor for AD [9, 10] as well as in mice chronically intoxicated with cocaine [11]. Mitovesicles, which were recently identified in our laboratory, originate from mitochondria, with size similar to exosomes and are informative of the oxidative status of the cell and of mitochondrial metabolic dysfunctions, such as in DS [8].

EVs play physiological roles in the brain, maintaining brain homeostasis by eliminating intracellular unneeded compounds from the cells, modulating intercellular communication, contributing to signaling functions, and regulating immunity [12]. However, studies have proposed a pathogenic role for EVs in age-associated neurodegenerative diseases by their contribution to the propagation of misfolded or aggregated proteins, such as pathogenic forms of tau and amyloid β (Aβ) in AD [13–15] and α-synuclein in PD [16].

We sought to characterize EVs in the normal brain during aging and compare the aging changes in males and females. Aging is characterized by a gradual and continuous loss of physiological function over time. Age-related changes in the brain manifest as decline of functional capabilities, such as learning and memory, language, and visuospatial abilities [17]. At the cellular and molecular levels, the brain displays numerous hallmarks of aging, including impairment in molecular waste disposal, mitochondrial dysfunction, oxidative stress, aberrant neuronal network activity, glial cell activation and inflammation [18]. Such a deterioration in functions of the aging brain is associated with an increased susceptibility to neurodegeneration, resulting in aging being a major risk factor for neurodegenerative diseases like AD and PD. In age-associated neurodegenerative diseases, dysfunctional protein homeostasis during aging [19, 20] leads to the accumulation of misfolded proteins. Given the sex differences in brain physiology and structure as well as in the incidence of different neurodegenerative disorders, with higher incidence of AD and lower incidence of PD in women as compared to men, [21–24], we undertook to investigate differences in EVs in the brain of males and females.

The involvement of brain EVs in the pathogenesis of neurodegenerative disorders emphasizes the importance of understanding the biology of brain EVs during aging and the effect of sex. Thus, in this study, we investigated the effects of aging and sex on the levels of brain microvesicles, exosomes and mitovesicles. We show that while the amount of EVs positive for mitochondria-derived mitovesicle proteins increases during aging in brains of both sexes, the amount of microvesicles and ESCRT-dependent exosomes increases only in the brain of female mice.

## Materials and Methods

### Mice

C57BL/6 J female and male mice (Jackson laboratory, Bay Harbor, ME, USA, RRID:IMSR_JAX:000664) were studied at 3, 6, 12, 18, and 24 months of age. All animal procedures were performed following the National Institutes of Health (NIH) guidelines with approval from the Institutional Animal Care and Use Committee at the Nathan S. Kline Institute for Psychiatric Research. All experiments adhered to the ‘Animal Research: Reporting In Vivo Experiment’s (ARRIVE) guidelines.

### Brain EV Isolation

Isolation of brain EVs was performed as previously described [25, 26]. Briefly, frozen right hemibrains were treated with 20 units/ml papain (Worthington, Lakewood, NJ, USA) in Hibernate A solution (HA, 3.5 ml/sample; BrainBits, Springfield, IL, USA) for 15 min at 37 °C for gentle dissociation. The papain was inactivated by the addition of 6.5 ml of ice-cold HA supplemented with protease inhibitors [5 μg/ml leupeptin, 5 μg/ml antipain dihydrochloride, 5 μg/ml pepstatin A, 1 mM phenylmethanesulfonyl fluoride (PMSF), 1 μM E-64, all from Sigma-Aldrich, St. Louis, MO, USA]. The digested brain tissue was centrifuged at 300 *g* for 10 min at 4 °C to discard the cells, and the supernatant was sequentially filtered through a 40 μm mesh filter (BD Biosciences, San Jose, CA, USA) and a 0.2 μm syringe filter (Corning Life Sciences, Teterboro, NJ, USA). The filtrate was subjected to a sequential centrifugation at 4 °C, at 2000 *g* for 10 min and 10,000 *g* for 30 min to discard membranes and debris, and at 100,000 *g* for 70 min to pellet the EVs. To wash the EVs, the EV pellet was resuspended in 60 ml of PBS and centrifuged at 100,000 *g* for 70 min at 4 °C. The washed EVs were then resuspended in 2 ml of 0.95 M sucrose solution, which was inserted inside a sucrose step gradient column (six 2-ml steps starting from 2.00 M sucrose, then 1.65, 1.30, 0.95, 0.60, and 0.25 M sucrose, bottom to top). The sucrose step gradient was centrifuged at 200,000 *g* for 16 h at 4 °C, followed by the collection of fractions from the top of the gradient. The fractions were resuspended in PBS and then centrifuged at 100,000 *g* for 70 min, and three major fractions containing EVs (B, C and D) were collected. Aliquots for nanoparticle tracking analysis and transmission electron microscopy were then prepared, and the rest of the EV suspensions was lysed by adding 2X RIPA buffer (2% v/v Triton X-100, 2% w/v sodium deoxycholate, 0.2% w/v sodium dodecyl sulfate, 300 mM sodium chloride, 100 mM Tris–HCl pH 7.4, 2 mM ethylenediaminetetraacetic acid, all from Sigma-Aldrich) supplemented with protease inhibitors (5 μg/ml leupeptin, 5 μg/ml antipain, 5 μg/ml pepstatin A, 1 mM PMSF, 1 μM E-64, all from Sigma-Aldrich) for western blot analyses.

### Nanoparticle Tracking Analysis (NTA)

The resuspended EVs were characterized and quantified using the ZetaView model PMX-220 analyzer (Particle Metrix, Meerbusch, Germany) equipped with 488 nm and 640 nm lasers. Prior to each analysis, using deionized distilled water, the machine underwent a quality control analysis, followed by assessment of polystyrene particles of 100 nm size (Thermo Fisher Scientific, Waltham, MA, USA) with an expected range of 100 nm ± 3.9 nm. Prior to video capture of particles, the EVs were further diluted in filtered PBS. The dilution factors used for acquisition were 1:500,000 for fraction B, 1:400,000 for fraction C and 1:200,000 for fraction D. The parameters set for acquisition were: Sensitivity 85, Shutter 100, Frame rate 30, Minimum area 10, Maximum area 2000, Trace length 15, and Bin size 5. The EV particles were measured in each of the 11 video locations within a certain video capture time interval.

### Negative Stain of EVs and Transmission Electron Microscopy (TEM)

Brain EVs were fixed in a paraformaldehyde (PFA) solution (Electron Microscopy Sciences, Hatfield, PA, USA) containing 2% (v/v) PFA in a 100 mM sodium cacodylate buffer (Electron Microscopy Sciences). Formvar covered carbon coated copper 200 mesh grids (Ted Pella Inc, Redding, CA, USA) were glow discharged in a PELCO EasyGlow (Ted Pella). 3 μl of fixed EVs were added to the grid, held by self-clamping tweezers, and left for 20 s. The solution was then wicked off with a Whatman filter paper and negatively-stained in 1% w/v uranyl acetate (Electron Microscopy Sciences) before being immediately wicked off using filter paper. This process was repeated three times before a final incubation of 5 min. The excess solution was wicked off and the grid allowed to air dry. Imaging took place on a Talos L120C transmission electron microscope operating at 120 kV (Thermo Fisher Scientific) using a 16 M Ceta Camera.

### Western Blot Analysis

The total protein levels of brain homogenates and of isolated brain EVs were determined by using Pierce BCA (bicin-choninic acid) Protein Assay Kit (Thermo Fisher Scientific) according to the manufacturer’s protocol. Brain homogenates (10 μg) of left hemibrains or EV proteins (5 μg) isolated from the right hemibrains of the same mice were run on 4–20% tris–glycine gels (Criterion precast gel, Bio-Rad, Hercules, CA, USA) and transferred onto Immobilon-P PVDF membranes (EMD Millipore, Billerica, MA, USA). Membranes were then blocked using 5% (w/v) blotting grade non-fat dry milk (Bio-Rad) or 5% (w/v) BSA (Sigma-Aldrich) in Tris-buffered saline (50 mM Tris-base, 150 mM NaCl, pH 7.5) containing 0.05% (v/v) Tween-20 (Sigma-Aldrich). Blots were incubated with primary antibodies overnight at 4 °C or for 1.5–2 h at room temperature, followed by incubation with horseradish peroxidase (HRP)-conjugated secondary antibodies (Jackson ImmunoResearch, West Grove, PA, USA). Blots were developed with electrochemiluminescence (ECL) substrate (Pierce, Thermo Fisher Scientific) or femto ECL (Pierce, Thermo Fisher Scientific) when is necessary. Primary antibodies used were: β-tubulin I + II (1:5,000, Sigma-Aldrich Cat# T8535, RRID:AB_261795), Alix (1:1,000, Cell Signaling Technology Cat# 92880, Danvers, MA, USA, RRID:AB_2800192), Annexin A2 (1:5,000, Abcam Cat# ab178677, Cambridge, UK), CD63 (1:1,000, Abcam Cat# ab217345, RRID:AB_2754982), GM130 (1:1,000, BD Biosciences Cat# 610823, RRID:AB_398141), HSC70 (1:1,000, Santa Cruz Biotechnology Cat# sc-7298, Santa Cruz, CA, USA, RRID:AB_627761), Lamin A/C (1:100, Santa Cruz Biotechnology Cat# sc-376248, RRID:AB_10991536), OCIAD2 (1:1,000, Sigma-Aldrich Cat# HPA040979, RRID:AB_10795497), SFXN5 (1:40,000, Abcam Cat# ab172971), TSG101 (1:1,000, Proteintech Cat# 14497–1-AP, Rosemont, IL, USA, RRID:AB_2208090), VDAC (1:1,000, Cell Signaling Technology Cat# 4866, RRID:AB_2272627). Protein bands were quantified using ImageJ (NIH, Bethesda, MD, USA) [27]. To investigate the aging effect on EV subtypes in each sex, EV markers were normalized to the loading volume, to the brain weight, and to the total sum of the bands in each blot for the quantification of the level of vesicles positive for each EV marker.

### Statistical Analyses

Data are presented as mean ± SEM (standard error of the mean). NTA data were analyzed by performing one-way ANOVA, followed by Bonferroni’s post hoc multiple comparisons, using the software GraphPad Prism 6 (version 6.01, GraphPad Holdings, San Diego, CA, USA). Changes in the levels of brain EVs in female and male mice with age in western blot analyses were analyzed by performing one-way ANOVA, followed by Bonferroni’s post hoc multiple comparisons. The same statistical analyses were performed to compare the difference in the level of expression of EV proteins in brain homogenates during aging from both females and males. The *p*-values are given for all statistical analyses.

## Results

### Characterization of EVs Isolated From the Brains of Female and Male Wild-Type Mice at Different Ages

**Fig. 1:**
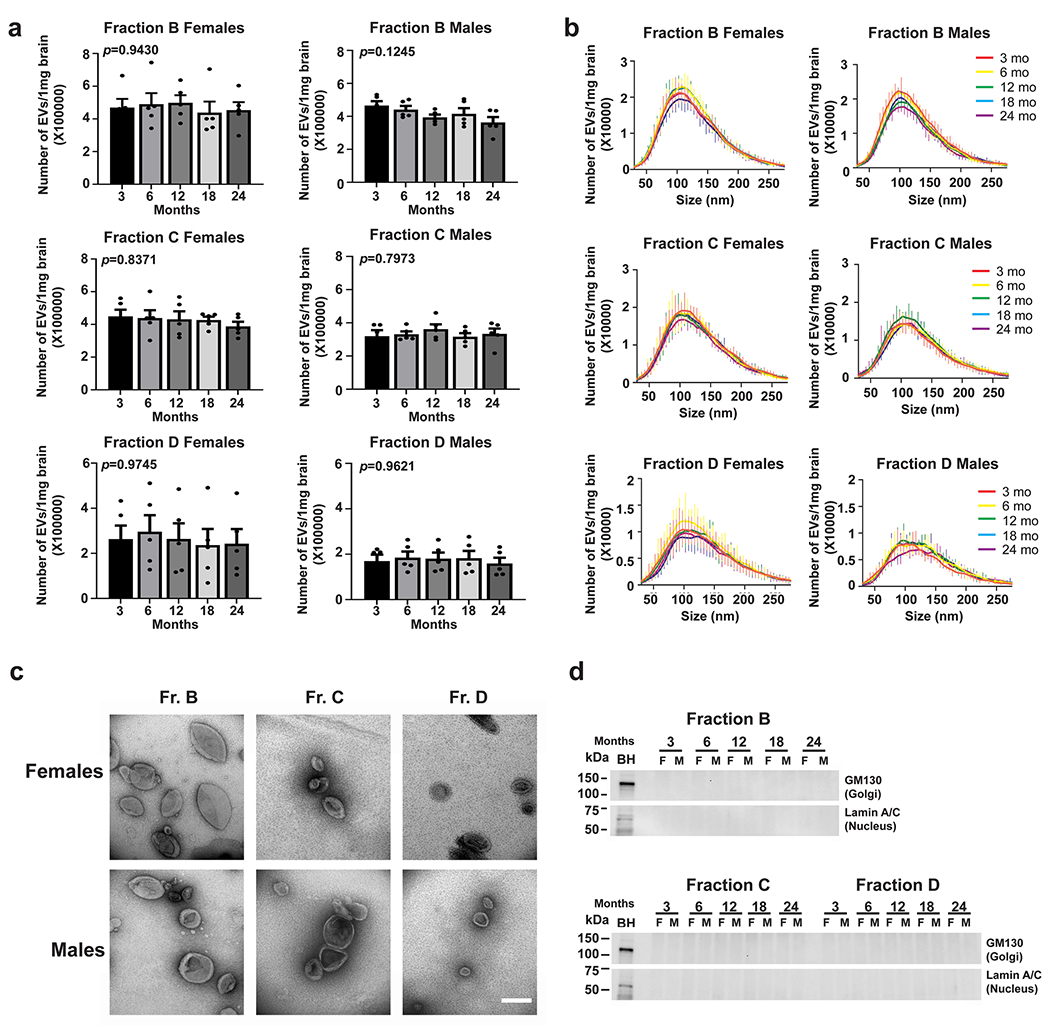
Isolation of brain EVs—their number, size, morphology and purity. **a** Representative nanoparticle tracking analysis (NTA) of EVs (fractions B, C and D) isolated from the brains of female and male mice at 3, 6, 12, 18, and 24 months of age show no significant difference in the number of particles with age. The number of particles was normalized to brain weight. **b** Representative profiles of size distribution of EVs (fractions B, C and D) isolated from the brains of female and male mice at 3, 6, 12, 18, and 24 months of age were determined by NTA. The number of particles was normalized to brain weight. The figure indicates the mode of size distribution. **c** Representative electron photomicrographs of EVs isolated from the brains of a male and a female mice at 18 months of age display typical cup-shaped EV morphology. Scale bar: 200 nm. **d** Representative western blot anlayses of intracellular proteins (GM130 and Lamin A/C) present in brain homogenates (BH) reveal their absence in EVs, confirming the purity of the isolated brain EVs. F: Female, M: Male. One-way ANOVA, *n* = 5 independent experiments

EVs were isolated simultaneously from the right hemibrains of mice at different ages (3, 6, 12, 18, and 24 months of age) using the sucrose step gradient ultracentrifugation method previously described [25, 26]. The number and size distribution of EVs were determined by NTA, which revealed no significant age-dependent changes in both females and males (Fig. 1a and 1b). To confirm that the EVs were not contaminated by debris or intracellular content, we examined the three fractions we have previously shown to contain EVs (B, C, and D) by TEM. TEM confirmed the isolation of EVs with a typical cup-shape without contamination of intracellular vesicles or debris. No apparent age- or sex-associated differences in EV morphology were detected (Fig. 1c). Western blot analyses using antibodies to proteins of intracellular organelles (GM130, Golgi; and Lamin A/C, nucleus) [28] showed that neither of these proteins were present in the EV fractions, (Fig. 1d), consistent with TEM showing lack of contamination by intracellular compartments.

### Sex Differences in the Levels of Brain Microvesicles and Exosomes with Aging

**Fig. 2:**
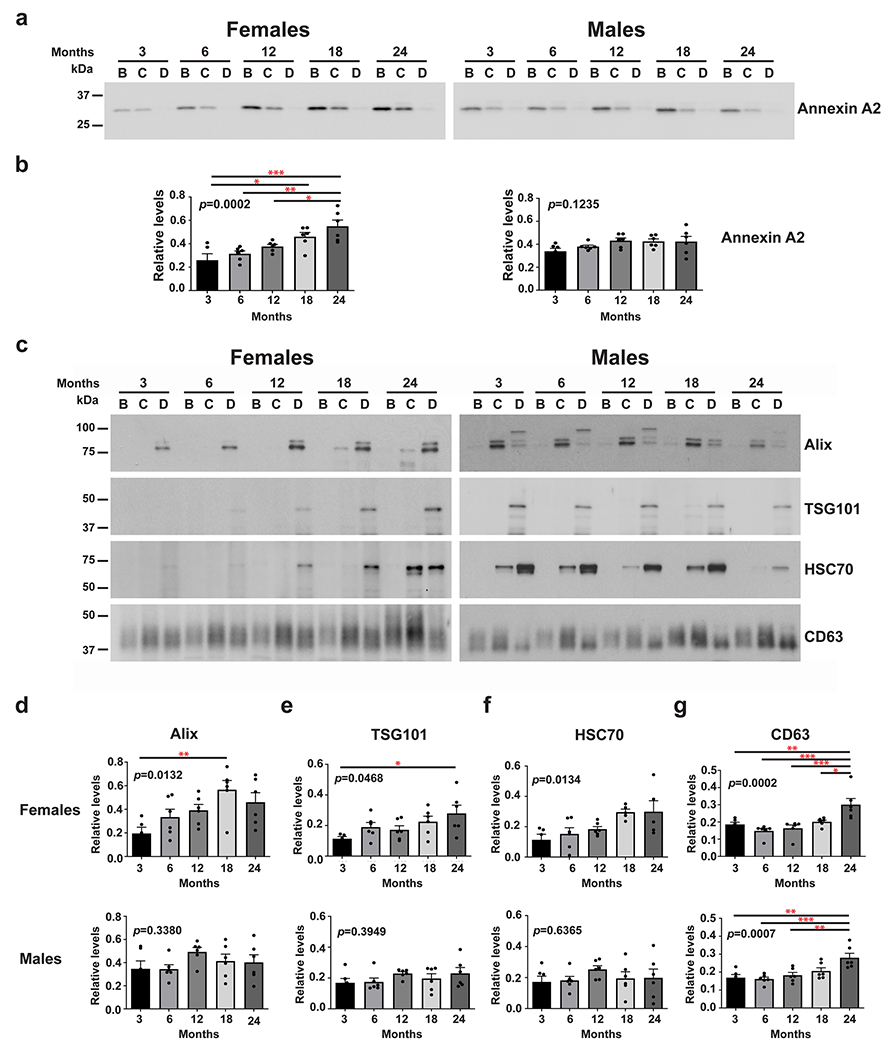
Microvesicle and exosome levels increase with age in the brain of females but not of males. **a** Representative western blot analysis of EVs isolated from the brains of female and male mice with an antibody to Annexin A2, a microvesicle marker, at 3, 6, 12, 18, and 24 months of age. **b** Quantification of the Annexin A2 bands reveals an age-associated increase only in females but not in males. **c** Representative western blot analyses of EVs isolated from the brains of female and male mice with antibodies to exosomal Alix, TSG101, HSC70 and CD63, at 3, 6, 12, 18, and 24 months of age. Quantifications of the exosome makers, Alix **d**, TSG101 **e**, and HSC70 **f** show age-associated increases only in females but not in males, except for the level of CD63 **g**, a tetraspanin protein, that increases with age in both females and males. One-way ANOVA, *n* = 6 independent experiments, * *p* < 0.05; ** *p* < 0.01; *** *p* < 0.001

Western blot analyses were used to investigate the levels of microvesicles and exosomes in the brains of female and male mice at each of the five age groups. The level of microvesicles, which are derived from the plasma membrane, was determined using an antibody to Annexin A2 [28] (Fig. 2a). Quantification of the bands shows that the Annexin A2 levels significantly increased with age in the brains of female (the effect of aging was significant at *p* = 0.0002) but not in the brains of male (*p* = 0.1235) mice (Fig. 2b), suggesting that aging only impacts the brain levels of microvesicles in females. We then analyzed proteins involved in exosome biogenesis. Western blot analyses revealed an age-associated increase in the levels of two components of the endosomal sorting complex required for transport (ESCRT) machinery, Alix and TSG101, involved in exosome biogenesis [29] in the brains of female (the effect of aging was significant at *p* = 0.0132 and 0.0468, respectively) but not male (*p* = 0.3380 and 0.3949, respectively) mice (Fig. 2c, 2d, and 2e). We also found that the level of the exosome intraluminal marker HSC70 [11] significantly increased with age only in the brains of female mice (female: *p* = 0.0134; male: *p* = 0.6365) (Fig. 2c and 2f). These data suggest an increase in the level of exosomes in the extracellular space through aging only in the brain of females. Exosomes can also be produced by an ESCRT-independent pathway involving tetraspanins such as CD63 [30, 31]. Our data show that the level of CD63 in brain EVs significantly increased with age in both females and males (the effect of aging was significant at *p* = 0.0002 and 0.0007, respectively) (Fig. 2c and 2g).

### Age-Dependent Changes in the Level of Mitovesicles

**Fig. 3:**
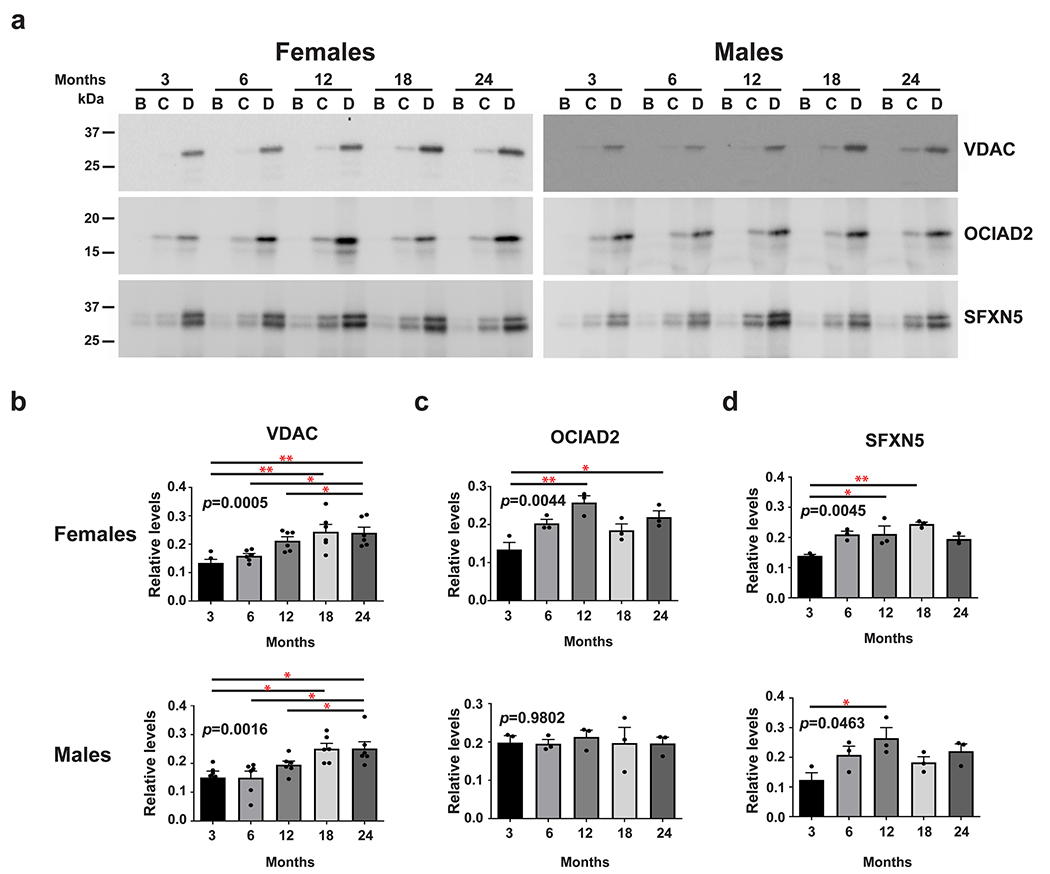
The levels of brain EVs positive for mitovesicle proteins secreted by astrocytes are affected by age in both females and males, and those secreted by neurons change with age only in females. **a** Representative western blot anlayses of EVs isolated from the brains of female and male mice with antibodies to the mitochondrial proteins VDAC, OCIAD2, and SFXN5 at 3, 6, 12, 18, and 24 months of age. Quantifications of the VDAC **b** and the astrocytic protein SFXN5 **d** bands reveal age-associated increases in both females and males while quanitification of the neuronal OCIAD2 **c** band shows an age-associated increase only in females but not in males. One-way ANOVA, *n* = 6 independent experiments for VDAC, *n* = 3 independent experiments for SFXN5 and OCIAD2, * *p* < 0.05; ** *p* < 0.01

Mitovesicles are a recently characterized type of EVs derived from mitochondria [8]. To determine whether mitovesicle levels change in the brain with aging and in a sex-dependent fashion, isolated EVs were subjected to western blot analysis using an antibody to the mitochondria-derived mitovesicle marker, VDAC [8]. We found that VDAC levels increase with aging in both sexes (*p* = 0.0005 for female *p* = 0.0016 for male) (Fig. 3a and 3b), suggesting that the level of mitovesicles increased with age in the brains of both females and males. Given that VDAC is a general marker of mitochondria and mitovesicles, this suggests a broad increase in mitovesicles within the brain during aging. To examine which cells in the brain are the source of increased levels of mitovesicles during aging, we examined by western blot analyses age-associated changes in the levels of cell-type-specific mitochondrial proteins [32]. We found that the level of SFXN5, an astrocytic mitovesicle protein, increased in the brains of both aged females and males (*p* = 0.0045 and 0.0463, respectively) (Fig. 3a and 3d). However, the level of the neuronal mitovesicle marker OCIAD2 increased with aging only in the brain of female mice (*p* = 0.0044) (Fig. 3a and 3c), suggesting neuronal-specific sex difference in mitovesicle secretion into the brain extracellular space with age.

### No Sex Differences and Age-Dependent Changes in the Level of Expression of EV Proteins in Brain Homogenates

**Fig. 4:**
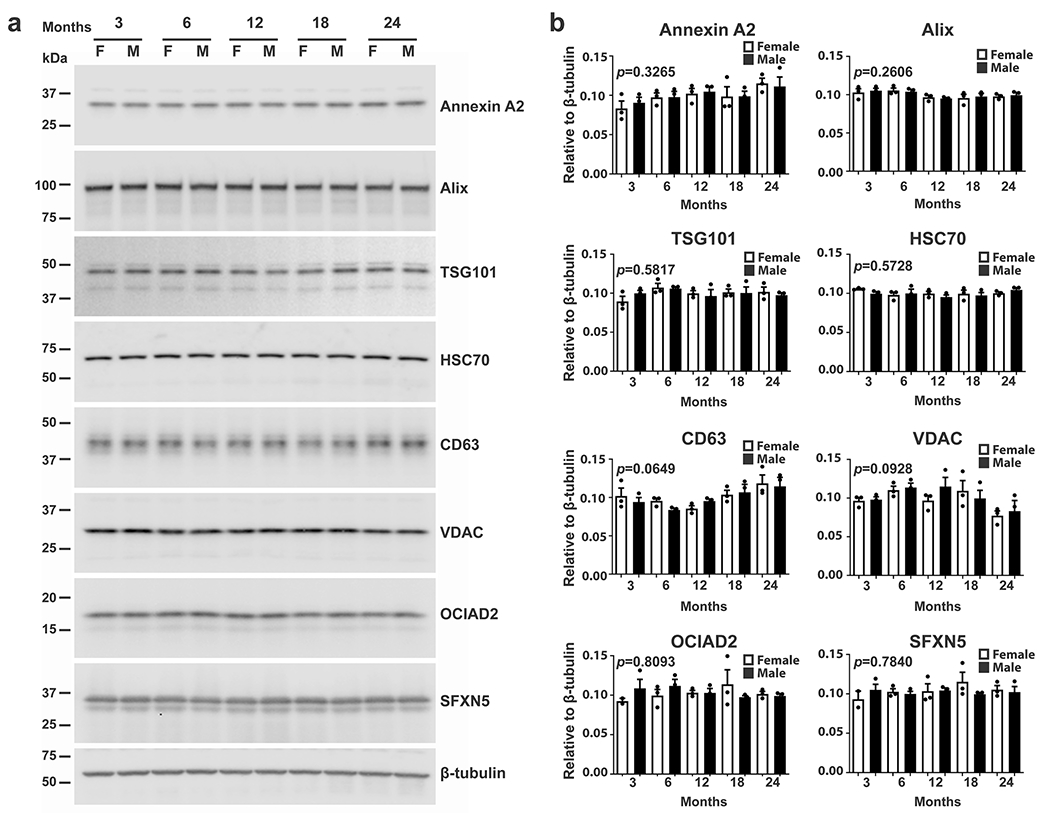
Expression levels of EV proteins in the brain are not affected by age and sex. **a** Representative western blot analyses of brain homogenates of female and male mice show the levels of the EV markers, Annexin A2, Alix, TSG101, HSC70, CD63, VDAC, OCIAD2, and SFXN5, and the loading control, β-tubulin, at 3, 6, 12, 18, and 24 months of age. **b** Quantification of the bands, normalized to β-tubulin, reveals no age-dependent change in both females and males. One-way ANOVA, *n* = 3 independent experiments

In order to investigate whether the sex difference in age-dependent increases in brain EVs is caused by changes in the level of expression of the proteins in brains of females and males, western blot analyses were performed using total brain homogenates from female and male mice. The data revealed neither sex difference nor age effect on the protein levels of the microvesicle (Annexin A2), exosome (Alix, TSG101, HSC70, and CD63), and mitovesicle (VDAC, OCIAD2, and SFXN5) proteins (Fig. 4a and 4b).

These data indicate that there are no sex differences in the expression of EV marker proteins, but a sex discrepancy exists in age-associated changes in the level of specific subtypes of brain EVs. While the amount of microvesicles, most types of exosomes, and neuronal-derived mitovesicles increases in an age-dependent manner only in the brain of female mice, the level of astrocytic-derived mitovesicles increases in the brains of both females and males.

## Discussion

This study shows that the secretion of specific types of EVs into the extracellular space significantly increases in the brain of female mice during aging but not in male mice. While we found that the secretion of brain exosomes positive for TSG101 and Alix increased during aging only in female brains, a sex-independent increase in brain exosomes positive for a tetraspanin protein, CD63, was found. Exosome generation is mediated by machineries involving ESCRT proteins such as TSG101 and Alix (an ESCRT-dependent pathway) and ceramide and/or tetraspanins (an ESCRT-independent pathway) throughout the endosomal pathway [33]. Our data thus demonstrate that the generation of subtypes of brain exosomes is differentially affected by sex during aging. In addition to a difference in exosome number, an age-associated increase in the level of microvesicles was found only in the brain of females. Thus, female-specific mechanisms are upregulated during aging: the production of exosomes through the ESCRT proteins-mediated machinery and the plasma membrane budding. Given that microvesicles and exosomes originate from the plasma membrane and the endosomal pathway, respectively, these data suggest sexual dimorphism in both cell membrane turnover and vesicle flux through the endosomal pathway.

Several studies of EVs in the circulation support our findings of an aging-dependent increase in EV secretion. A study that investigated mouse plasma EVs has shown an age-related decrease in lipoproteins but an increase with age of specific types of EVs using western blot analysis [34]. Given that the plasma particles analyzed by NTA contain lipoproteins, the finding by another study by NTA of an age-associated decrease of particle numbers in human and rodent plasma may be affected by an age-related decrease in lipoproteins [35]. Similarly, the particles analyzed by NTA in our study may contain lipoproteins. Thus, our western blot data showing significant increase in EV markers with age in combination with the quantification of EVs by NTA suggest that while lipid particles decrease during aging, the amount of specific EVs positive for EV markers increases. In addition, the particles analyzed by NTA contain a highly heterogenous population of EVs, of which only specific subtypes are affected by aging, contributing to the absence of age-dependent changes in the total number of brain EVs, determined by NTA. Aging neurons in culture were found to have higher number of ILVs in the lumen of MVBs and secrete smaller EVs than younger neurons [36]. RNA sequencing of genes expressed in the prefrontal cortex of young and aged male rats found an upregulation of transcripts linked to extracellular exosomes [37]. In a previous study, it was shown that the expression of exosome biogenesis genes in the mouse brain is affected by aging in a cell-type specific manner. While an increased expression of CD63 with age was demonstrated for most brain cells, including astrocytes, microglia, oligodendrocytes and pericytes, neurons have decreased expression of CD63 [38].

Advancing age is associated with a decline in the efficiency of exchange between the subarachnoid CSF and the brain parenchyma and it was proposed that impaired glymphatic clearance contributes to accumulation of Aβ in the brain, leading to cognitive decline among the elderly [39]. While it is not known if there is a sex difference in this phenomenon, lower clearance may also affect the level of EVs in the brain extracellular space. Higher number of senescent cells and senescence-associated increase in EV secretion were described, including in the brain, but this has been controversial, potentially due to different cells studied and technical procedures [40, 41]. To our knowledge, our study is the first to investigate the effect of aging on brain EVs, on different subtypes of EVs, and the difference between females and males, showing a female-specific increase in the levels of microvesicles and subtypes of brain exosomes with age. However, given that the EVs were isolated from whole hemibrains, it remains to be determined whether there are cell-type specific or region-specific sex-dependent differences in EV release with age.

An increased secretion of brain EVs likely has beneficial effects on brain function. The role of EV secretion was originally described as a complementary process to the lysosomal and proteasomal degradative pathways to eliminate obsolete membrane and cytosolic proteins during reticulocyte maturation [42]. Moreover, the lipid bilayer of EVs confers them stability in the extracellular environment, compared to secreted, soluble proteins [43]. This attribute supports longer survival of EV-associated neuroprotective proteins, such as cystatin C [44] in the extracellular space, extending the EV-associated protective proteins’ availability for neighboring cells following their EV uptake. Our data suggest that such EV-mediated beneficial effects achieved by the removal of intracellular waste and delivering neuroprotective proteins to adjacent cells in the brain are differentially regulated in the brain of females and males. This represents a sex difference in the ability of the brain to address age-related deficits in membrane trafficking pathways and preserve brain homeostasis.

While the beneficial effects of enhanced secretion of brain EVs have been proposed, a pathogenic role for EVs in neurodegenerative disorders has been suggested, given that proteins involved in neuropathogenesis are transferred via EVs. Specifically, it was demonstrated that in age-related neurodegenerative diseases, EVs harbor misfolded, unfolded, aggregated proteins. These include phosphorylated tau [45], Aβ oligomers [14, 15, 46] and α-synuclein preformed fibrils [47, 48]. Studies also demonstrated the uptake by cells of the secreted EVs containing the misfolded and aggregated neurotoxic proteins in vitro and in vivo, highlighting the EV-mediated transmission of pathology. Moreover, pharmacological inhibition of EV secretion was found to dramatically suppress the transmission of these proteins in rodent models of AD and PD [16, 49]. Given that sex, in addition to aging, is an established risk factor for AD, where females have higher incidence compared to males [21, 22], the age-associated increase in secretion of brain microvesicles and exosomes into the extracellular space only in females suggest that EVs contribute to AD progression in female brains.

Mitochondrial dysfunctions linked to normal brain aging were demonstrated by several studies, showing age-related alterations including impairment in the electron transport chain [50, 51], altered mitochondrial dynamics [52], and defective mitophagy [53, 54]. Our laboratory recently identified a novel subset of brain EVs derived from mitochondria, mitovesicles, with an increase in their secretion both in vitro into the culture media and in vivo into the brain extracellular space when mitochondrial dysfunctions occur in a mitophagy-independent fashion [8]. Here, we identified a sex-independent increase in the level of EVs positive for the mitovesicle marker, VDAC, and in the level of EVs positive for the astrocyte-derived mitochondrial protein SFXN5 during aging in the rodent brains. However, a female-specific increase in the level of the neuronal-derived OCIAD2 with age was found. Given that secretion of brain mitovesicles appears to be involved in the biology of mitochondrial regulation and dysfunction, our findings showing the increase in the levels of the brain mitovesicle during aging may reflect aging-dependent alterations in mitochondrial function in brains of both females and males, being more prominent in female brains.

An age-dependent increase in mitovesicle secretion into brain extracellular space may suggest that mitovesicle secretion is a marker of mitophagy-independent dysfunction, or alternatively, that mitophagy is compromised with aging and enhanced mitovesicle release is a compensatory mechanism. Thus, higher level of neuronal-derived mitovesicles in the brain of females as compared to males may suggest that neuronal mitochondria are more vulnerable in females. We suggest that similar to a beneficial role for exosome release to dysfunctional neuronal endosomal pathway, the release of mitovesicles by neurons with mitochondria dysfunction may be a compensatory mechanism that contributes to homeostasis in the female neuron.

The endosomal and mitochondrial systems are a point of vulnerability with aging and in many neurodegenerative disorders. Investigating EVs in brain, we find aging and sex differences, that may be an informative tool on brain health. However, it remains to be determined whether higher release of EVs is beneficial to the cells or contributes to pathology. Higher release of EVs may reveal higher level of pathology of the endosomal as well as mitochondrial systems, and/or transport of toxic material to other brain cells, propagating pathology, resulting in sex-dependent susceptibility to age-related neurodegenerative diseases. Alternatively, higher levels of microvesicles, exosomes, and neuronal mitovesicles in the female brain during aging may maintain neuronal homeostasis, a compensation mechanism that impacts successful brain aging.

In conclusion, we report here the sexual dimorphism in the amount of brain EVs secreted into brain extracellular space during aging, showing that the levels of plasma membrane-derived microvesicles, endosomal-derived exosomes, and neuronal mitochondria-derived mitovesicles are particularly enhanced in the brain of females as compared to males as they age. Our findings establish a baseline in the control brain, and provide an accessible, informative, and useful tool to investigate neurodegenerative and neurodevelopmental changes that differently affect males and females.

## Data Availability

All data generated or analyzed during this study are included in this published article. This study did not generate new unique reagents. No custom code was used in this study. No large datasets were generated.
